# Low Levels of Granulocytic Myeloid-Derived Suppressor Cells May Be a Good Marker of Survival in the Follow-Up of Patients With Severe COVID-19

**DOI:** 10.3389/fimmu.2021.801410

**Published:** 2022-01-28

**Authors:** Carlos Jiménez-Cortegana, Flora Sánchez-Jiménez, Antonio Pérez-Pérez, Nerissa Álvarez, Alberto Sousa, Luisa Cantón-Bulnes, Teresa Vilariño-García, Sandra Fuentes, Salomón Martín, Marta Jiménez, Antonio León-Justel, Luis de la Cruz-Merino, José Garnacho-Montero, Víctor Sánchez-Margalet

**Affiliations:** ^1^Department of Laboratory Medicine, Virgen Macarena University Hospital, Seville, Spain; ^2^Department of Medical Biochemistry and Molecular Biology, School of Medicine, University of Seville, Seville, Spain; ^3^Intensive Care Unit, Virgen Macarena University Hospital, Seville, Spain; ^4^Medical Oncology Service, Virgen Macarena University Hospital, Seville, Spain

**Keywords:** SARS-CoV2, COVID-19, ICU, MDSCs, Tregs, PD-1, OX40

## Abstract

Infection with severe acute respiratory syndrome coronavirus 2 (SARS-CoV-2) causes a disease (coronavirus disease 2019, COVID-19) that may develop into a systemic disease with immunosuppression and death in its severe form. Myeloid-derived suppressive cells (MDSCs) are inhibitory cells that contribute to immunosuppression in patients with cancer and infection. Increased levels of MDSCs have been found in COVID-19 patients, although their role in the pathogenesis of severe COVID-19 has not been clarified. For this reason, we raised the question whether MDSCs could be useful in the follow-up of patients with severe COVID-19 in the intensive care unit (ICU). Thus, we monitored the immunological cells, including MDSCs, in 80 patients admitted into the ICU. After 1, 2, and 3 weeks, we examined for a possible association with mortality (40 patients). Although the basal levels of circulating MDSCs did not discriminate between the two groups of patients, the last measurement before the endpoint (death or ICU discharge) showed that patients discharged alive from the ICU had lower levels of granulocytic MDSCs (G-MDSCs), higher levels of activated lymphocytes, and lower levels of exhausted lymphocytes compared with patients who had a bad evolution (death). In conclusion, a steady increase of G-MDSCs during the follow-up of patients with severe COVID-19 was found in those who eventually died.

## Introduction

Severe acute respiratory syndrome coronavirus 2 (SARS-CoV-2) infection may produce a systemic disease termed COVID-19 (coronavirus disease 2019), with high morbidity and mortality. This viral infection became a pandemic in 2020 and showed rapid and uncontrolled expansion worldwide in 2021, despite vaccination of around 50% of the total population. In fact, the COVID-19 pandemic is the major global health threat in the last century. Understanding of the pathophysiology of this viral infection is a major challenge and is absolutely necessary to improve the somber prognosis of COVID-19 patients with severe disease who require admission to the intensive care unit (ICU) (1).

Impairment of both innate and adaptive immunity has been described in patients with SARS-CoV-2 infection, and it has been associated with poor outcomes (2). Lymphopenia is a frequent finding in these patients and has been identified as a variable independently associated with mortality (3). It has been observed that lymphocyte subsets such as CD4^+^ T cells, CD8^+^ T cells, B cells, and natural killer (NK) cells decreased in COVID-19 patients, especially in severe cases. Moreover, the underlying mechanisms responsible for lymphopenia in COVID-19 patients still need to be investigated since these could be responsible for the delayed virus clearance and the increased mortality rate among patients. In line with this notion, myeloid-derived suppressor cells (MDSCs) are a heterogeneous group of immature myeloid cells that mainly inhibit T-cell immune responses and NK cell proliferation using different mechanisms. They consist of monocytic (M-MDSCs) and granulocytic (G-MDSCs) subsets, which have been recently defined as pathologically activated neutrophils and monocytes with potent immunosuppressive activity (4).

The role of MDSCs was first discovered in cancer patients, but they have been found to be important in several disease processes such as sepsis (5). The persistence of these cells may contribute to long-lasting immunosuppression, thus leaving patients unable to resolve infections. We have recently found increased M-MDSCs in patients with mild COVID-19 (6), suggesting that the monocytic MDSC subset may contribute to lymphopenia and immune suppression in COVID-19. Nevertheless, the role of MDSCs in the pathogenesis of severe COVID-19 has not yet been fully elucidated, although recent studies have reported that MDSCs might influence both disease severity and mortality (7, 8). Moreover, the levels of M-MDSCs have been recently found to predict the severity of COVID-19 (9), whereas others have found an expansion of G-MDSCs in patients with severe COVID-19 (8). Moreover, the function and transcriptome of G-MDSCs may explain, at least in part, the severity of the disease (10). In line with this, MDSCs have been proposed as a potential biomarker and a therapeutic target in this viral infection (11). MDSCs are also well known to induce regulatory T cells (Tregs), which are a specialized subpopulation of T cells that can inhibit T-cell proliferation and cytokine production. Patients with COVID-19 exhibit low levels of circulating Tregs, being lower in severe cases, although this study did not include patients admitted to the ICU (12). We also found decreased levels of Tregs in patients with mild COVID-19 (6).

In addition, programmed death-1 (PD-1) binds to its ligands (PD-L1 or PD-L2), expressed on antigen-presenting cells (APCs), to generate inhibitory signals that downregulate T-cell-mediated immune responses. Lymphocytes from COVID-19 patients have been found to have increased expressions of inhibitory molecules, such as PD-1 or CTLA-4, producing an ineffective immune response (13, 14). Upregulation of PD-1 on CD4^+^ T cells in SARS-CoV-2 patients has also been associated with poor outcomes at 30 days (15).

Therefore, information about the behavior of the lymphocyte subsets in critically ill COVID-19 patients is lacking or has been obtained from a reduced number of patients. The present study explored the immunosuppressive cell populations, MDSCs, and Tregs in critically ill COVID-19 patients and compared their evolutions in patients who died and those who survived.

## Materials and Methods

### Study Design

This is a prospective, observational, cohort study that enrolled critically ill adult patients (age ≥ 18 years) with COVID-19 admitted to the ICU of the Virgen Macarena University Hospital (Seville, Spain) from October 2020 to March 2021. The exclusion criteria were as follows: patients with previous immunosuppression (solid organ or hematologic transplantations, hematologic malignancies, or taking immunosuppressants before hospital admission) and pregnant women.

The following data were noted: age, gender, body mass index (BMI), comorbidities (diabetes mellitus, liver cirrhosis, chronic renal disease, chronic heart failure, and chronic obstructive pulmonary disease), disease chronology (time from the onset of symptoms and from hospital admission to ICU admission), pharmacological treatments, ICU length of stay (LOS), and ICU mortality. Illness severity at ICU admission was assessed using the Acute Physiology and Chronic Health Evaluation (APACHE) II score and the Sequential Organ Failure Assessment (SOFA) scale, considering the worst data point of the first 24 h in the ICU (16, 17). Nosocomial infections included ventilator-associated pneumonia, primary bacteremia, and catheter-related bloodstream infection that were diagnosed following current definitions (18). Septic shock was diagnosed following the Sepsis-3 criteria (19). Continuous renal replacement therapy was initiated by the attending physician and followed the recommendations of the Spanish Society of Intensive Care Medicine (20).

### Patients

We studied the immunological characteristics of peripheral blood cells from 80 COVID-19 patients hospitalized in the ICU with respiratory failure and positive for real-time reverse transcriptase polymerase chain reaction (RT-PCR) (Allplex 2019-nCoV Assay; Seegene, Seoul, South Korea) assay for nasal and pharyngeal swab specimens. Blood was obtained in the first 24 h following admission into the ICU using samples sent to the hospital laboratory for routinary tests and weekly thereafter up to death from ICU discharge. The study was approved by the Institutional Review Board (ref. MDSC-Treg_COVID-19, code no. 0908-N-20) according to the ethical principles included in the Declaration of Helsinki 1964 (2013 update). Written consent was not required.

### Flow Cytometry Analysis in Whole Blood Samples

Cell populations (MDSCs, Tregs, and both OX40^+^PD-1^−^ and PD-1^+^OX40^−^ T cells, as well as CD4 T cells, CD8 T cells, and total T, B, and NK cells) were measured by flow cytometry using the FACSCanto II flow cytometry system (Becton Dickinson, Franklin Lakes, NJ, USA) from EDTA-K3 tubes. Analyses were carried out from ICU admission to the last determination before ICU discharge or death. Furthermore, the total lymphocyte, monocyte, and granulocyte counts were obtained from hematologic counts (Sysmex CS-1000).

M-MDSCs were gated as CD45^+^CD11b^+^CD33^+^HLA-DR^low/−^CD14^+^CD15^−^, G-MDSCs as CD45^+^CD11b^+^CD33^+^HLA-DR^low/−^CD14^−^CD15^+^, Tregs as CD4^+^CD25^high^CD127^low/−^, activated T cells as CD3^+^CD4^+^OX40^+^PD-1^−^ and CD3^+^CD8^+^OX40^+^PD-1^−^, and exhausted T cells as CD3^+^CD4^+^PD-1^+^OX40^−^ and CD3^+^CD8^+^PD-1^+^OX40^−^, as previously described (6, 21). Total T, B, and NK cells were gated as CD3^+^, CD19^+^, and CD16^+^CD56^+^, respectively. CD4 and CD8 T cells were gated as CD3^+^CD4^+^ and CD3^+^CD8^+^, respectively. The absolute cell number was calculated by multiplying the percentages obtained from flow cytometry with the total leukocyte count obtained from the hematologic count. Total MDSCs were calculated as the sum of the M-MDSC and G-MDSC counts, total activated T cells as the sum of the CD3^+^CD4^+^OX40^+^PD-1^−^ and CD3^+^CD8^+^OX40^+^PD-1^−^ T-cell counts, and total exhausted T cells as the sum of the CD3^+^CD4^+^PD-1^+^OX40^−^ and CD3^+^CD8^+^PD-1^+^OX40^−^ T-cell counts.

### Monoclonal Antibodies

The following antibodies were obtained from Becton Dickinson Immunocytometry Systems (San Jose, CA, USA) and were used at the manufacturer’s recommended concentrations.

MDSCs: PerCP-Cy5.5 mouse anti-human CD 45 (ref. no. 564105), PE mouse anti-human CD 33 (ref. no. 555450), APC-Cy7 rat anti-CD11b (ref. no. 557657), PE-Cy7 mouse anti-human HLA-DR (ref. no. 560651), FITC mouse anti-human CD 14 (ref. no. 555397), and APC mouse anti-human CD 15 (ref. no. 551376).

Tregs: human regulatory T-cell cocktail (ref. no. 560249), including FITC anti-human CD4, PE-Cy7 anti-human CD25, and Alexa Fluor 647 anti-human CD127.

Activated and inhibited T cells: APC-Cy7 mouse anti-human CD3 (ref. no. 561800), PE-Cy7 mouse anti-human CD4 (ref. no. 557852), PerCP-Cy5.5 mouse anti-human CD8 (ref. no. 565310), FITC mouse anti-human OX-40 (CD134; ref. no. 555837), and APC mouse anti-human PD-1 (CD279) (ref. no. 558694).

T, B, and NK cells and CD4 and CD8 T cells: Multitest 6-Color TBNK (ref. no. 644611).

### Data Analysis

Statistical analysis was performed and graphs were constructed using GraphPad Prism 8.0.2 (GraphPad Software, San Diego, CA, USA). Continuous variables were shown as the median and 95% confidence intervals. Qualitative variables were presented as absolute numbers and percentages. Normal distribution of the analyzed variables was examined using a histogram, box plot, the Q–Q plot, and the outcomes of the Kolmogorov–Smirnov normality test.

Non-parametric tests were used due to the absence of normality. The Mann–Whitney *U* test was used to compare the cell distributions between the discharged and deceased COVID-19 patients. Wilcoxon’s test was used to compare the cell distributions in each group of patients at ICU admission *vs.* the last determination. The Friedman test and Bonferroni corrections were performed to compare the cell distributions in each group of patients during the ICU follow-up from admission to the third week of stay. Bivariate correlations among cell populations were carried out using Spearman’s coefficient. *P*-values ≤0.05 were considered statistically significant differences.

## Results

### Clinical Characteristics of COVID-19 Patients

Eighty-seven patients diagnosed of COVID-19 and hospitalized in the ICU during the study period were screened, but seven patients were excluded (three patients with onco-hematologic diseases, two renal transplant patients, and two patients taking immunosuppressant drugs for systemic diseases). Thus, 80 patients were analyzed. The age of the patients was 62 years (median, p25–p75= 59–66 years). The male/female ratio of COVID-19 patients was 76.5%/23.5%. The patients’ clinical characteristics are shown in **Table 1**. Thirty-eight patients were discharged from the ICU alive, but two of them died in the hospital.

**Table 1:** Clinical characteristics of COVID-19 patients.

Characteristics	*N* (%)
Patients	80 (100.00)
Age (years)^a^	62 (59–66)
Female sex	16 (23.75)
Comorbidities	
COPD	11 (13.75)
Chronic heart failure	7 (8.75)
Cancer	3 (3.75)
Chronic kidney disease	1 (1.25)
Liver cirrhosis	1 (1.25)
Diabetes	17 (21.25)
Body mass index (kg/m^2^)^a^	29.40 (28.50–31.10)
APACHE II^a^	10 (8–11)
SOFA score^a^	4 (4–4)
Mechanical ventilation (at any time in the ICU)	52 (65.00)
Treatment	
Corticosteroids	80 (100.00)
Tocilizumab	10 (12.50)
CRRT	6 (7.50)
ECMO	7 (8.75)
Complications in ICU	
Nosocomial infection	43 (53.75)
Septic shock	23 (28.75)
Acute renal failure	20 (25.00)
ICU mortality	38 (47.50)
Hospital mortality	40 (50.00)
90-day mortality	39 (48.75)

COPD, chronic obstructive pulmonary disease; SOFA, Sequential Organ Failure Assessment; APACHE, Acute Physiology and Chronic Health Evaluation; CRRT, continuous renal replacement therapy; ECMO, extracorporeal membrane oxygenation.

a Data shown as median and 95% confidence intervals.

### Circulating MDSCs in COVID-19 Patients at Admission and During the ICU Stay

**Figure 1:**
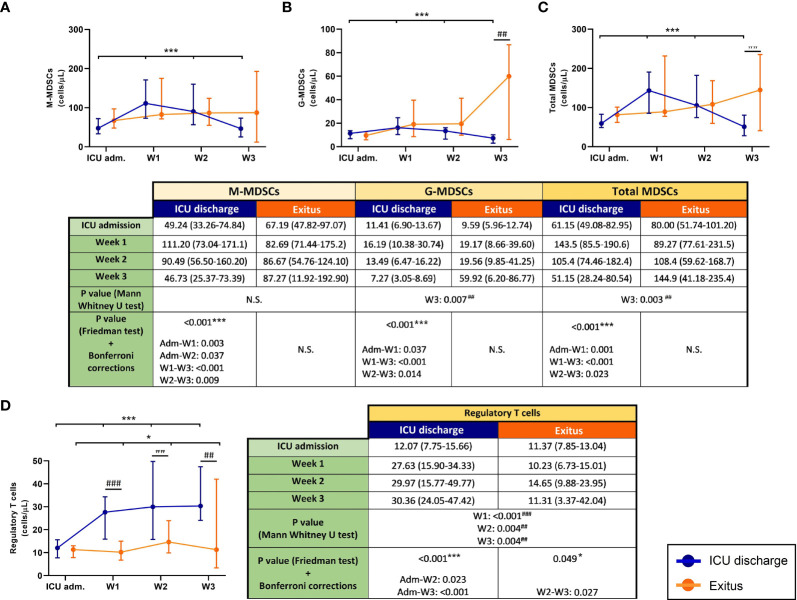
Circulating myeloid-derived suppressive cells (MDSCs) and regulatory T cells (Tregs) in discharged (*blue*) and deceased (*red*) coronavirus disease 2019 (COVID-19) patients during their follow-up in the ICU. **(A)** Monocytic MDSCs. **(B)** Granulocytic MDSCs. **(C)** Total MDSCs. **(D)** Tregs. All data shown are the median and 95% confidence intervals of cells per microliter. ^##^*p* ≤ 0.01, ^###^*p* ≤ 0.001 comparing opposite groups, respectively; *p ≤ 0.05, ***p ≤ 0.001 compared with ICU admission, respectively. ns, not significant.

The follow-up of blood MDSCs from severe COVID-19 patients in the ICU is shown in **Figures 1A–C**. All MDSC populations significantly decreased (Friedman test: *p* < 0.001) in patients who were discharged from the ICU, whereas MDSCs increased in those who passed away. Between both groups of patients, the results of the Mann–Whitney *U* test revealed statistically significant differences in G-MDSCs and total MDSCs at the last determination (*p* = 0.007 and *p* = 0.003, respectively).

**Figure 2:**
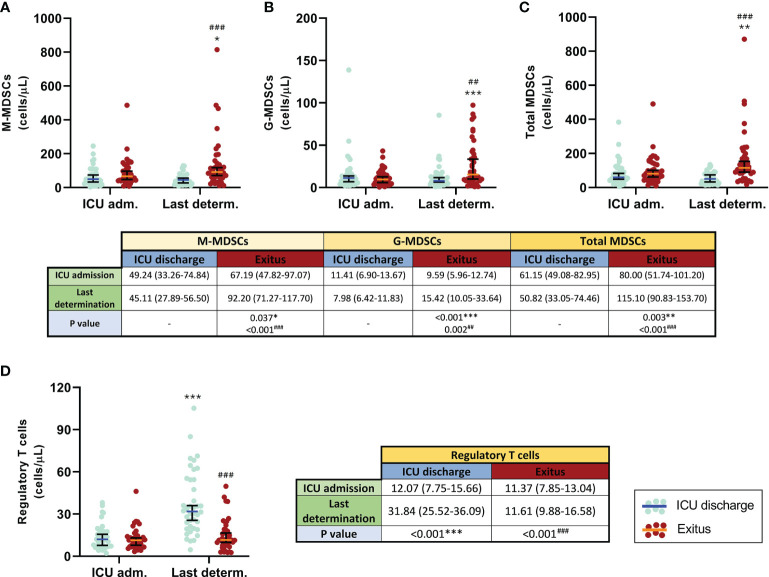
Comparison of circulating myeloid-derived suppressive cells (MDSCs) and regulatory T cells (Tregs) in discharged (*blue*) and deceased (*red*) coronavirus disease 2019 (COVID-19) patients between the first and the last determination in the ICU. **(A)** Monocytic MDSCs. **(B)** Granulocytic MDSCs. **(C)** Total MDSCs. **(D)** Tregs. All data shown are the median and 95% confidence intervals of cells per microliter. ^##^*p* ≤ 0.01, ^###^*p ≤* 0.001 comparing opposite groups, respectively; *p ≤ 0.05, **p ≤ 0.01, ***p ≤ 0.001 compared with ICU admission, respectively.

Similar results were obtained when the first and the last determination for each patient were compared. In those who were discharged from the ICU, all MDSC subsets and total MDSCs slightly decreased (**Figures 2A–C**). In contrast, all MDSC populations were remarkably increased in patients who passed away (*p* = 0.037 for M-MDSCs, *p* < 0.001 for G-MDSCs, and *p* = 0.003 for total MDSCs); in consequence, significant differences were found between the patient groups at the last determination (*p* < 0.001 for both M-MDSCs and total MDSCs and *p* = 0.002 for G-MDSCs), also shown in **Figures 2A–C**.

Both M-MDSC and G-MDSC populations were also positively correlated at the beginning (*r*_S_ = 0.296, *p* = 0.007) and at the end (*r*_S_ = 0.326, *p* = 0.003) of their stay in the ICU.

### Blood Tregs in COVID-19 Patients at Admission and During the ICU Stay

The trend of Tregs in both discharged and deceased patients during the follow-up (**Figure 1D**) was significant (*p* < 0.001 and *p* = 0.049, respectively). Although all patients had similar concentrations of Tregs in blood at ICU admission, the follow-up revealed different trends in both groups: Tregs from discharged patients had a 2.5-fold increase, whereas those from deceased patients remained practically constant. In addition, significant differences between patient groups were found from the first to the third week of their stay in the ICU (*p* < 0.001 in the first week and *p* = 0.004 in the second and third weeks).

Comparison of the first and the last determination (**Figure 2D**) showed that the levels of circulating Tregs were similar in all COVID-19 patients at ICU admission. However, this T-cell subset significantly increased in discharged patients (*p* < 0.001) and remained constant in the group of patients who finally died. Consequently, significant differences were also found between both groups at the last determination (*p* < 0.001).

### Concentrations of Exhausted T Cells in COVID-19 Patients at Admission and During the ICU Stay

**Figure 3:**
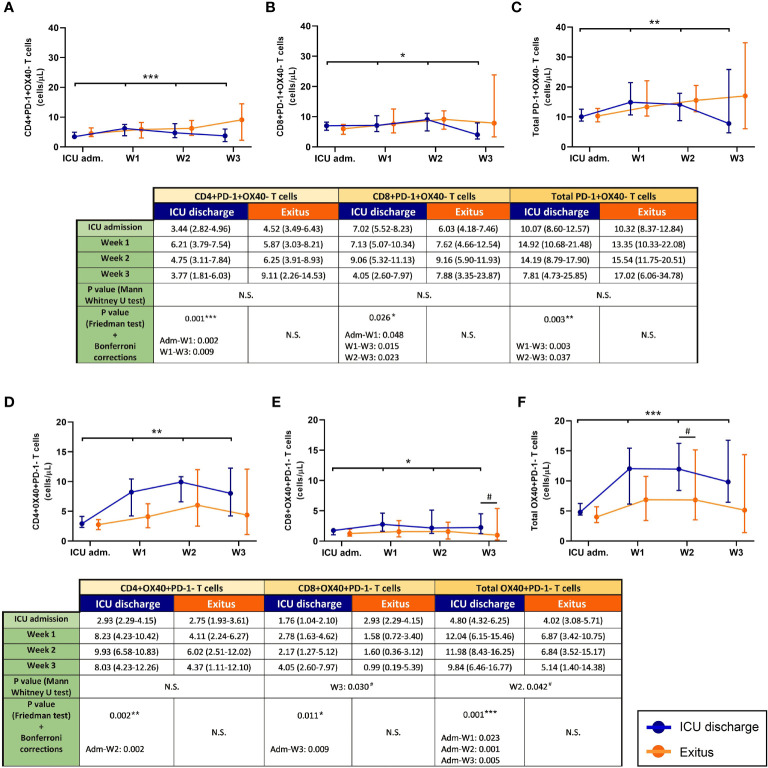
Circulating exhausted (PD-1^+^OX40^−^) and activated (OX40^+^PD-1^−^) T cells in discharged (*blue*) and deceased (*red*) coronavirus disease 2019 (COVID-19) patients during their follow-up in the ICU. **(A)** CD4^+^PD-1^+^OX40^−^ T cells. **(B)** CD8^+^PD-1^+^OX40^−^ T cells. **(C)** Total PD-1^+^OX40^−^ T cells. **(D)** CD4^+^OX40^+^PD-1^−^ T cells. **(E)** CD8^+^OX40^+^PD-1^−^ T cells. **(F)** Total OX40^+^PD-1^−^ T cells. All data shown are the median and 95% confidence intervals of cells per microliter. ^#^*p* ≤ 0.05 comparing opposite groups; *p ≤ 0.05, **p ≤ 0.01, ***p ≤ 0.001 compared with ICU admission, respectively. ns, not significant.

The evolution of exhausted (PD-1^+^OX40^−^) T cells during the follow-up of severe COVID-19 patients is shown in **Figures 3A–C**. As happened with MDSCs, there was a depletion of exhausted T cells (particularly from the first week after admission into ICU) in the discharged group (*p* = 0.001 for CD4^+^, *p* = 0.0026 for CD8^+^, and *p* = 0.003 for total T cells), whereas these T-cell subsets slightly increased during the follow-up in the group of patients who finally died.

**Figure 4:**
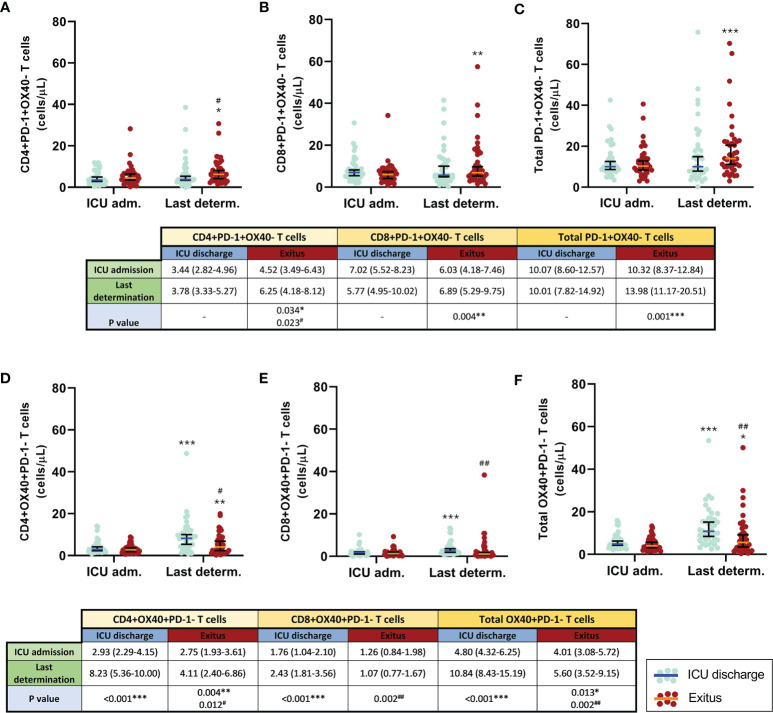
Comparison of the circulating exhausted (PD-1^+^OX40^−^) and activated (OX40^+^PD-1^−^) T cells in discharged (*blue*) and deceased (*red*) coronavirus disease 2019 (COVID-19) patients between the first and the last determination in the ICU. **(A)** CD4^+^PD-1^+^OX40^−^ T cells. **(B)** CD8^+^PD-1^+^OX40^−^ T cells. **(C)** Total PD-1^+^OX40^−^ T cells. **(D)** CD4^+^OX40^+^PD-1^−^ T cells. **(E)** CD8^+^OX40^+^PD-1^−^ T cells. **(F)** Total OX40^+^PD-1^−^ T cells. All data shown are the median and 95% confidence intervals of cells per microliter. ^#^*p* ≤ 0.05m, ^##^*p* ≤ 0.01 comparing opposite groups, respectively; *p ≤ 0.05, **p ≤ 0.01, ***p ≤ 0.001 compared with ICU admission, respectively.

When the first and the last blood determination were compared (**Figures 4A–C**), the levels of both exhausted CD4^+^ T cells and total T cells remained without significant changes; however, exhausted CD8^+^ T cells slightly decreased in patients who were discharged from the ICU, whereas all exhausted T-cell populations significantly increased in patients who passed away (*p* = 0.034 for CD4^+^, *p* = 0.004 for CD8^+^, and *p* = 0.001 for total T cells). Significant differences between groups were only found at the last determination of exhausted CD4 T cells (*p* = 0.023).

Positive correlations were found between the levels of CD4^+^ and CD8^+^ inhibited T cells at both the beginning (*r*_S_ = 0.255, *p* = 0.022) and the last analysis (*r*_S_ = 0.536, *p* < 0.001) of each patient.

### Circulating Activated T Cells in COVID-19 Patients at Admission and During the ICU Stay

During the follow-up (**Figures 3D–F**), only discharged patients presented a significant increment in the evolution of activated (OX40^+^PD-1^−^) T cells (*p* = 0.002 for CD4^+^, *p* = 0.011 for CD8^+^, and *p* = 0.001 for total T cells). Moreover, notable differences were also obtained when a comparative analysis between both patient groups was performed (*p* = 0.030 during the third week for CD8^+^ and *p* = 0.042 during the second week for total OX40^+^PD-1^−^ T cells).

In the comparison between the first and the last determination in the ICU (**Figures 4D–F**), it was observed that the concentrations of CD4^+^, CD8^+^, and total activated (OX40^+^PD-1^−^) T cells were significantly increased in patients with the best outcome (*p* < 0.001 in all cases). In deceased patients, the levels of activated CD4^+^ and total T cells also increased, but those of the cytotoxic T-cell subpopulation remained constant. At the last determination, notable differences were found between groups (*p* = 0.012 for CD4^+^ and *p* = 0.002 for CD8^+^ and total T cells).

In addition, strong positive correlations were found between helper and cytotoxic activated T cells in patients hospitalized in the ICU (*r*_S_ = 0.340, *p* = 0.0021) and during their last determination (*r*_S_ = 0.579, *p* = 0.0001).

### Tregs Were Positively Correlated With Activated T Cells and MDSCs With Exhausted T Cells in COVID-19 Patients in ICU

Apart from the significant positive correlations mentioned above, we also observed other strong correlations between the different cell populations. Tregs were positively correlated with OX40^+^PD-1^−^ T cells both at admission into ICU and at the end of the stay and were negatively correlated with G-MDSCs at the last determination, as shown in **Table 2**. For its part, G-MDSCs were positively correlated with CD8^+^ and total exhausted T cells at the moment of hospitalization and also with CD4^+^ after the stay in the ICU. Total MDSCs were only positively correlated with exhausted T cells at the end of admission. All statistically significant correlations between cell groups are shown in **Table 2**.

**Table 2:** Spearman’s correlations (*r*_S_) between cell populations.

	ICU admission		Last determination	
	*r*_S_	*p-*value	*r*_S_	*p-*value
G-MDSCs *vs.* M-MDSCs	0.296	0.0071*	0.326	0.0028*
** ***vs.* CD4^+^PD-1^+^OX40^−^ T cells	0.140	0.2113	0.526	0.0004*
** ***vs.* CD8^+^PD-1^+^OX40^−^ T cells	0.293	0.0081*	0.377	0.0014*
** ***vs.* total PD-1^+^OX40^−^ T cells	0.261	0.0184*	0.473	0.0009*
** ***vs.* Tregs	-0.013	0.9071	-0.323	0.0030*
Total MDSCs *vs.* CD4^+^PD-1^+^OX40^−^ T cells	0.088	0.4332	0.246	0.0271*
** ***vs.* CD8^+^PD-1^+^OX40^−^ T cells	0.126	0.2624	0.280	0.0114*
** ***vs.* total PD-1^+^OX40^−^ T cells	0.131	0.2453	0.296	0.0072*
CD4^+^PD-1^+^OX40^−^ T cells *vs.* CD8^+^PD-1^+^OX40^−^ T cells	0.340	0.0021*	0.579	0.0001*
CD4^+^OX40^+^PD-1^−^ T cells *vs.* CD8^+^OX40^+^PD-1^−^ T cells	0.255	0.0223*	0.536	0.0002*
Tregs *vs.* CD4^+^OX40^+^PD-1^−^ T cells	0.504	0.0002*	0.516	0.0003*
*vs.* CD8^+^OX40^+^PD-1^−^ T cells	0.285	0.0103*	0.520	0.0003*
*vs.* total OX40^+^PD-1^−^ T cells	0.489	0.0004*	0.557	0.0001*

MDSCs, myeloid-derived suppressive cells; G-MDSCs, granulocytic MDSCs; M-MDSCs, monocytic MDSCs; Tregs, regulatory T cells.

*Statistically significant differences.

## Discussion

SARS-CoV-2 infection causes immune defects such as lymphopenia (22) in patients with mild (6) and severe (23) COVID-19. Moreover, persistent lymphopenia was observed in patients with severe COVID-19 after 3 weeks of follow-up (24), but the lymphocyte reduction was more highlighted in critically ill patients, especially T lymphocytes (25). In our study, we found lymphopenia in all COVID-19 patients after admission into ICU, and the lymphocyte levels were increased in blood during the follow-up regardless of their outcomes (**Table 3**). At least in part, it could occur because the treatments contributed to the activation of the immune system of COVID-19 patients to fight the viral disease. However, 40 patients eventually died, suggesting that there are mechanisms of immunosuppression due to infection with SARS-Cov-2, as MDSCs could be. Accordingly, T cells, especially CD4^+^ and NK cells, were significantly lower in patients with fatal outcomes. As previously explained, MDSCs are pathologically activated neutrophils and monocytes with potent immunosuppressive activity (4), and they mediate the mechanism of immune downregulation, especially the inhibition of lymphocyte activation and proliferation (26). In line with this, it has been found that cells with MDSC features are implicated in COVID-19, and several reports have described the accumulation of potent immunosuppressive M-MDSCs and G-MDSCs in the disease (4, 27, 28). In fact, a high M-MDSC/monocyte ratio has been associated with secondary infections and death due to the disease (29); the granulocytic subset has also been associated with mortality in severe COVID-19 (7).

**Table 3:** Granulocyte, monocyte, and lymphocyte counts during the follow-up of severe COVID-19 patients.

Cell populations	Patient status	ICU admission	Week 1	Week 2	Week 3
Granulocytes	Discharge	9,000 (8,540–10,200)	11,135 (10,310–15,510)	9,800 (8,320–11,550)	9,150 (5,930–17,580)
	Death	9,600 (8,400–10,990)	13,770 (11,410–16,630)	11,240 (9,040–13,920)	10,980 (5,620–19,310)
Monocytes	Discharge	360 (270–460)	930 (660–1,180)	860 (630–940)	600 (410–1,130)
	Death	410 (340–1,180)	520 (350–720)	490 (320–750)	520 (330–750)
Total lymphocytes	Discharge	830 (610–940)	1,520 (1,040–1,980)	1,440 (1,020–1,830)	1315 (890–1,610)
	Death	690 (520–820)	640 (470–820)	785 (660–1050)	940 (600–1,520)
T cells	Discharge	414 (336–593)	988 (616–1,314)	1,094 (708–1,269)	947 (632–1,187)
	Death	361 (263–458)	400 (320–504)	537 (471–755)	705 (366–1,533)
CD4 T cells	Discharge	226 (168–380)	734 (370–932)	663 (521–966)	723 (444–874)
	Death	219 (163–249)	299 (222–349)	388 (290–515)	429 (218–610)
CD8 T cells	Discharge	128 (102–155)	258 (162–342)	177 (139–379)	202 (155–564)
	Death	100 (78–154)	94 (52–138)	128 (88–178)	172 (54–289)
B cells	Discharge	168 (122–226)	308 (222–439)	264 (145–384)	218 (145–438)
	Death	144 (107–173)	143 (113–198)	172 (134–230)	192 (85–255)
NK cells	Discharge	112 (70–130)	95 (68–129)	104 (69–145)	119 (70–187)
	Death	96 (58–137)	54 (40–75)	66 (37–127)	65 (58–150)

Cell counts (in cells per microliter) are presented as the median and 95% confidence intervals.

NK cells, natural killer cells.

We aimed to analyze MDSCs, namely, the monocytic (M-MDSCs) and granulocytic (G-MDSCs) subsets, and the lymphocyte subpopulations in patients with severe COVID-19 from admission into the ICU and during the follow-up until discharge or death. MDSC expansion has been related to dysfunction in lymphocytes (30), and MDSCs have even been proposed as potential biomarkers and therapeutic targets in COVID-19 (11). Nevertheless, the increase in MDSC levels could not be a specific mechanism of immunosuppression in COVID-19 since it has also been described in other viral infections, such as influenza (31, 32), hepatitis B, hepatitis C, and human immunodeficiency virus (33).

Even though we found increased levels of MDSCs in all patients on the first day after admission into the ICU compared with control subjects and patients with mild COVID-19 (6), there were no differences between patients with good evolution (discharged from the ICU) and those who died in the ICU. Nevertheless, the follow-up of patients showed that those with good evolution (discharged) had lower levels of MDSCs, especially G-MDSCs. The relative influence of MDSC subtypes is not clear. M-MDSCs have been found accumulated in severe COVID-19 patients, and they seem to have been responsible for the production of IL-6 in these patients (34), whereas others have found that G-MDSCs may predict fatal COVID-19 outcomes (7, 35). Our data were similar and showed that the number of circulating G-MDSCs may predict fatal outcomes only at the weekly follow-up. It is important to mention that most of the G-MDSC data at week 3 for the deceased patients were collected on the same day of death, or at 1 or 2 days before death as maximum, so the peak value of circulating G-MDSCs in these patients (~60 cells/μl) may be considered as a “danger point” to predict death, even more if we consider the notable differences regarding the levels of G-MDSCs obtained during the follow-up (~10, 19, and 20 cells/μl at ICU admission and during the first and second weeks, respectively), as shown in **Figure 1B**.

MDSCs are known to mediate the production of Tregs (36); conversely, Tregs are known to regulate MDSCs (37). The crosstalk between both cell types has been previously studied (38). However, we found decreased numbers of circulating Tregs in severe COVID-19 patients, although they have increased circulating MDSCs. We already discovered this discrepancy in mild COVID-19 patients (6), and we assumed that the lymphopenic effect of SARS-CoV-2 infection may also affect Tregs. In fact, other research groups have also found decreased numbers of Tregs in COVID-19 patients (12), especially in critically ill patients (39). In line with this, we have also found that patients with better outcomes have increased numbers of circulating Tregs, probably due to the recovery of total lymphocytes.

In any case, the increased numbers of MDSCs seemed to be sufficient, at least in part, to account for the higher numbers of exhausted T cells in COVID-19 patients with fatal outcomes. In line with this, an increase in exhausted T cells (expressing PD-1) has previously been found in COVID-19 patients, which showed a relationship with their clinical outcomes, suggesting that the expression of PD-1 on T cells may be a risk factor for unfavorable outcomes in these patients (40). Moreover, we have found a positive correlation between the numbers of MDSCs and exhausted T cells in patients with severe COVID-19 admitted into the ICU.

Moreover, lower numbers of both activated CD4^+^ and CD8^+^ T cells were found at the last determination in patients who died in the ICU compared with patients who were discharged from the ICU. These data suggest that the increase in MDSCs could help prevent T-cell activation, therefore further contributing to the immunosuppression in severe COVID-19 patients with fatal outcomes.

One limitation of the study is the inclusion of a small number of patients from just one center. Nevertheless, the high ICU mortality rate of COVID-19 allowed us to study a similar number of deceased patients and patients who were discharged from the ICU, even though these patients were prospectively recruited.

In conclusion, patients with severe COVID-19 admitted into the ICU had increased levels of MDSCs and exhausted T cells, whereas they had decreased circulating Tregs and activated T cells. However, only the weekly follow-up of these cellular populations could differentiate the group of patients with good outcomes (ICU discharge) from those who eventually passed away, who had increased numbers of MDSCs, especially the granulocytic subset, which may be an interesting biomarker of fatal outcomes in the follow-up of severe COVID-19 patients admitted into the ICU.

## Data Availability Statement

The raw data supporting the conclusions of this article will be made available by the authors, without undue reservation upon reasonable request.

## Ethics Statement

The studies involving human participants were reviewed and approved by IRB Virgen Macarena and Virgen Rocio University Hospital. Written informed consent for participation was not required for this study, in accordance with the national legislation and the institutional requirements.

## Author Contributions

LC-M, JG-M, and VS-M designed the work. JG-M, NA, AS, and LC-B acquired clinical data. CJ-C, FS-J, AP-P, TV-G, SF, SM, MJ, AL-J participated in the management of samples and all authors participated in the analysis of data. CJ-C, LC-M, JG-M and VS-M drafted the work and all the authors revised it critically for important intellectual content.

## Funding

This work was funded in part by Funds from Junta de Andalucía (PAIDI group CTS-151).

## Conflict of Interest

The authors declare that the research was conducted in the absence of any commercial or financial relationships that could be construed as a potential conflict of interest.

## Publisher’s Note

All claims expressed in this article are solely those of the authors and do not necessarily represent those of their affiliated organizations, or those of the publisher, the editors and the reviewers. Any product that may be evaluated in this article, or claim that may be made by its manufacturer, is not guaranteed or endorsed by the publisher.
